# Study on the Effect of Polyamine Water Treatment Agent on Metal Corrosion Inhibition in Boiler Steam–Water System

**DOI:** 10.3390/ma17051063

**Published:** 2024-02-26

**Authors:** Zhijuan Zhao, Bingqing Cao, Bo Zhao, Sheng Chen, Dong Jin

**Affiliations:** Key Laboratory of Special Equipment Safety and Energy-Saving for State Market Regulation, China Special Equipment Inspection & Research Institute, Beijing 100029, China; 13126584199@163.com (B.C.); zhaobo@csei.org.cn (B.Z.); chensheng_csei@163.com (S.C.)

**Keywords:** boiler steam–water system, corrosion of carbon steel, polyamine water treatment agent, film-forming property, electrochemical property, corrosion inhibition

## Abstract

A polyamine water treatment agent was prepared with the film-forming amine (N-oleyl-1,3-propylenediamine) and the neutralizing amine (cyclohexanamine) under optimal conditions. The copper sulfate liquid drop experiment showed that a protective film was formed by the polyamine water treatment agent on carbon steel. The analyses of the polarization curve and electrochemical impedance spectroscopy of carbon steel indicated that the polyamine water treatment agent exhibited geometric effects, which could inhibit both anode and cathode reactions of carbon steel, and the corrosion inhibition effect of the polyamine water treatment agent showed an extreme-concentration phenomenon. A metal corrosion experiment in a simulated boiler steam–water system indicated that the polyamine water treatment agent mitigated the corrosion of carbon steel at different temperatures, and the corrosion inhibition rates of the polyamine water treatment agent in liquid and gas environments at 150 °C were 53.84% and 67.43%, respectively, better than that at 350 °C. SEM-EDS characterization indicated that the formation of the corrosion product, iron oxide, on the carbon steel was reduced with the addition of the polyamine water treatment agent in the simulated boiler steam–water system.

## 1. Introduction

Water is the basic constituent required to ensure the normal operation of a boiler system. For the economically viable and environmentally responsible operation of a boiler, it is essential to recycle as much of the water as possible in all of the boiler system. This is particularly challenging in high-pressure steam–water systems, since the impurities in water result in metal corrosion, which is dangerous. Therefore, the use of water treatment agents is necessary to ensure the safe and reliable operation of boiler steam–water systems.

Ammonium hydroxide, hydrazine and sodium phosphate are traditional boiler water treatment agents that can regulate boiler water quality to reduce metal corrosion in boiler steam–water systems and are widely applied in many enterprises [1,2]. However, the properties of these traditional water treatment agents limit their further use [3,4,5]. For example, ammonium hydroxide and hydrazine pose a threat to human safety. Sodium phosphate cannot form a passivation film on the inner wall of boilers under high-temperature and high-pressure steam–water systems, and phosphate scale forms easily with iron ions in water. Moreover, the national requirements for phosphorus emissions have become more stringent and the use of sodium phosphate has been further restricted. Therefore, the use of traditional water treatment agents is constrained.

Polyamine water treatment agents with film-forming amines as the main component have been used to inhibit metal corrosion in boiler steam–water systems in many industrial applications [6,7,8,9], since they can form a protective film which acts as a hydrophobic barrier against corrosive species, such as oxygen and carbonic acid [10,11,12,13]. In one study, a film-forming amine was defined by the general formula R_1_-[NH-(R_2_)-]_n_-NH_2_, where n is an integer between 0 and 7, R_1_ is an unbranched alkyl chain with 12 to 18 carbon atoms and R_2_ is a short-chain alkyl group that usually contains 1 to 4 carbon atoms [14]. Because of its effectiveness in inhibiting corrosion while maintaining a clean heat transfer surface, this polyamine water treatment agent is increasingly employed as an alternative to traditional water treatment agents [15,16]. In addition to film-forming amines, polyamine water treatment agents typically contain neutralizing amines such as cyclohexanamine, 3-methoxypropylamine and 3-methoxypropylamine to control the pH and act as hydrotropes for poorly soluble film-forming amines [17,18]. Although boiler steam–water systems have already been treated with polyamine water treatment agents [19,20,21,22], their behavior and inhibition mechanisms are still not sufficiently well understood to ensure their optimal use [14,23].

In this work, a polyamine water treatment agent was prepared using the film-forming amine N-oleyl-1,3-propylenediamine and the neutralizing amine cyclohexanamine, and optimal preparation conditions were obtained. The film-forming property of the prepared polyamine water treatment agent on carbon steel was characterized with the copper sulfate liquid drop experiment. The corrosion inhibition mechanism of the polyamine water treatment agent on carbon steel was studied using electrochemical characterization including polarization curves and electrochemical impedance spectra. The corrosion inhibition effect of the polyamine water treatment agent on metal in a boiler steam–water system was evaluated by a metal corrosion experiment in a high-temperature and high-pressure autoclave. Moreover, the surface micromorphology and elemental compositions of the corroded carbon steel under different conditions were characterized with scanning electron microscope–energy dispersive X-ray spectrometry to further explore the corrosion inhibition effect of the polyamine water treatment agent.

## 2. Materials and Methods

### 2.1. Materials

N-oleyl-1,3-propylenediamine (OLDA) was sourced from Aladdin (Shanghai, China) and cyclohexylamine (CHA) from Alfa (Shanghai, China). Amounts of 40 mL of 0.4 mol/L CuSO_4_ solution, 20 mL of 10% NaCl solution and 1.5 mL of 0.1 mol/L HCL solution were added to a volumetric bottle and diluted to 100 mL with pure water to prepare an acidic aqueous solution of copper sulfate. The carbon steel was a 20# carbon steel sample of 50 mm × 25 mm × 2 mm.

### 2.2. The Preparation of Polyamine Water Treatment Agent

A total of 1 g of OLDA was added and dissolved in 2 g of CHA; then, 100 mL of pure water was added and stirred for 30 min at room temperature to prepare the polyamine water treatment agent. The polyamine water treatment agent was prepared by changing the adding sequence of raw materials, OLDA concentration and OLDA/CHA ratio.

### 2.3. The Film-Forming Property Characterization

The 20# carbon steel sample was soaked in absolute ethyl alcohol for 5 min and wiped clean with degreasing cotton, and then was blotted with filter paper and placed in a dryer for more than 4 h for later use. The carbon steel sample was hung in the solution with different concentrations of the polyamine water treatment agent for 24 h, and the stirring speed of the solution was 500 r/min. Then, the carbon steel sample was taken out and blotted with filter paper. The carbon steel sample was placed on the table horizontally, and a drop of the acidic aqueous solution of copper sulfate was dropped onto the surface of the carbon steel sample. The moment the droplet fell onto the surface of the carbon steel sample, we started timing with a stopwatch. When the droplet on the surface of the carbon steel sample began to change from blue to red, the timing was stopped. The discoloration time of the copper sulfate liquid drop was obtained and recorded to characterize the film-forming property of the polyamine water treatment agent. The result was the average value of 10 measurements.

### 2.4. The Electrochemical Property Characterization

The polarization curves and electrochemical impedance spectra (EIS) of the carbon steel sample were measured in solution with 200 mg/L NaCl and different concentrations of the polyamine water treatment agent. The carbon steel sample was encapsulated with epoxy resin with an exposure area of 1 cm × 1 cm, and then was polished with a sandpaper step by step. Finally, the carbon steel sample was cleaned with ethanol and dried with absorbable cotton, and then was used as the working electrode. The platinum sheet was used as the auxiliary electrode and the saturated calomel electrode was used as the reference electrode. The potential sweep interval was ±250 mV (relative to the open circuit potential) and the scanning rate was 1 mV/s during polarization curve measurement. EIS was measured under the open circuit potential, the frequency range was 100 kHz~0.02 Hz and the disturbance potential was 5 mV.

### 2.5. The Metal Corrosion Experiment

The 20# carbon steel sample was soaked in absolute ethanol for 5 min and wiped clean with degreasing cotton, and then was blotted with filter paper and placed in a dryer for more than 4 h for later use. Then, 1000 mL of aqueous solution with different concentrations of the polyamine water treatment agent was poured into a autoclave. Six carbon steel samples were taken out from the dryer and weighed as m. Three weighed carbon steel samples were hung on the bottom of the sample rack and the other three were hung on the top of the sample rack and then placed in the autoclave. The autoclave was closed and nitrogen was aerated into the solution to deoxygenate for 1 h. The autoclave was then heated to the set-point temperature, maintained at the set-point temperature for 24 h and thereafter, the carbon steel samples were taken out after the autoclave returned to room temperature. The carbon steel samples were cleaned with a brush and soaked in pickling solution for 10 min, rinsed with pure water and blotted with filter paper. Then, the carbon steel samples were immersed in anhydrous ethanol for 5 min, blotted with filter paper and put in the dryer for more than 4 h. Finally, the carbon steel samples were taken out from the dryer and weighed as m_0_.

The corrosion weight loss rate ν (mm/a) was expressed as:

$$\nu =\frac{8760\times \left(m-m_{0}\right)\times 10}{s\times \rho \times t}$$

where m was the initial weight of the carbon steel and the unit of m was g; m_0_ was the weight of the corroded carbon steel after cleaning and the unit of m_0_ was g; s was the surface area of the carbon steel and the unit of s was cm^2^; ρ was the density of the carbon steel and the unit of ρ was g/cm^3^; t was the time of a metal corrosion experiment and the unit of t was h; 8760 was the total hours one year and the unit was h/a and 10 represented 10 mm equivalent to 1 cm and the unit was mm/cm.

The corrosion inhibition efficiency η (%) of film-forming amine on carbon steel was expressed as:

$$\eta =\frac{\nu_{0}-\nu_{1}}{\nu_{0}}\times 100\%$$

where ν_0_ was the corrosion weight loss rate of the carbon steel in pure water and the unit of ν_0_ was mm/a and ν_1_ was the corrosion weight loss rate of the carbon steel in aqueous solution with polyamine water treatment agent and the unit of ν_1_ was mm/a.

### 2.6. The Micromorphology Characterization

The surface micromorphology of carbon steel was characterized with a scanning electron microscope (SEM) and the acceleration voltage and the current were 5 kV and 10 μA. The surface element distribution of carbon steel was characterized by energy dispersive X-ray spectrometry (EDS) and the acceleration voltage and the current were 20 kV and 10 μA.

## 3. Results and Discussion

To solve the problem that OLDA could not be dissolved in water, CHA was used as a kind of hydrotrope to dissolve OLDA; then, pure water was added to prepare the polyamine water treatment agent. The concentration of the polyamine water treatment agent was expressed in terms of OLDA concentration. The polyamine water treatment agent with 10 g/L of OLDA and the OLDA/CHA ratio 1:2 was prepared at room temperature in the same way as the boiler water treatment agent studied in our previous work [24].

### 3.1. The Film-Forming Property of Polyamine Water Treatment Agent

**Figure 1:**
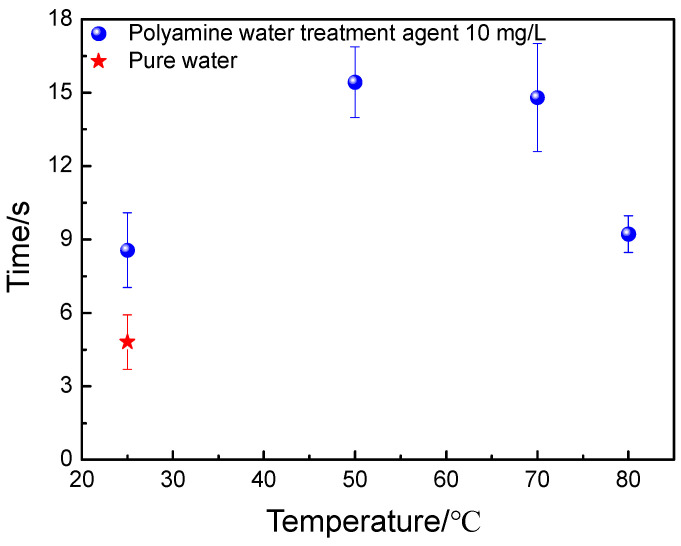
The discoloration times of the copper sulfate liquid drops on the surface of the carbon steel after being dipped in pure water and aqueous solution with 10 mg/L of polyamine water treatment agent at different temperatures.

The discoloration times of the copper sulfate liquid drops on the surface of the carbon steel after being dipped in the pure water and aqueous solution with 10 mg/L of polyamine water treatment agent at different temperatures are shown in Figure 1. The discoloration time of the copper sulfate liquid drop on the surface of the carbon steel after being dipped in pure water at room temperature was 4.81 s, indicating the poor corrosion resistance of the carbon steel. When the carbon steel was dipped in aqueous solution with 10 mg/L of polyamine water treatment agent, the discoloration time of the copper sulfate liquid drop on its surface increased significantly. This indicated that a protective film was formed on the surface of the carbon steel to improve the corrosion resistance of carbon steel. Moreover, the formation of the protective film by the polyamine water treatment agent on the surface of the carbon steel was significantly affected by the solution temperature. The discoloration time of the copper sulfate liquid drop on the surface of the carbon steel was 8.57 s at room temperature. When the solution temperature was 50, 70 and 80 °C, the discoloration times of the copper sulfate liquid drop on the surface of the carbon steel were 15.43, 14.80 and 9.22 s, respectively. The discoloration time of the copper sulfate liquid drop on the surface of the carbon steel increased at first, and then decreased with the solution temperature increasing according to the results, indicating that the corrosion resistance of the carbon steel was improved at first, and then declined but was still superior to the original carbon steel.

**Figure 2:**
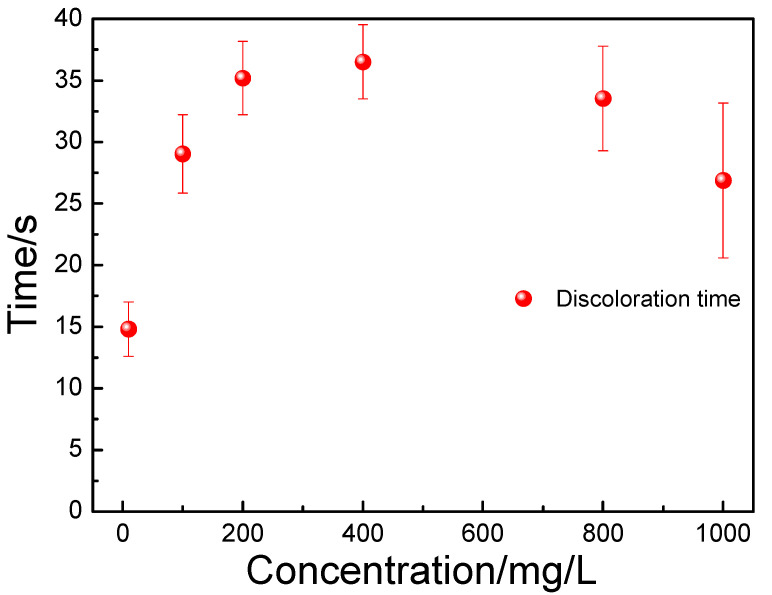
The discoloration times of the copper sulfate liquid drop on the surface of the carbon steel after being dipped in aqueous solution with different concentrations of polyamine water treatment agent.

The discoloration times of the copper sulfate liquid drops on the surface of the carbon steel after being dipped in aqueous solution with different concentrations of the polyamine water treatment agent are shown in Figure 2. When the concentration of polyamine water treatment agent increased from 10 to 100 mg/L, the discoloration time of the copper sulfate liquid drop on the surface of the carbon steel exhibited a significant increase and increased from 14.80 to 29.04 s. When the concentration of polyamine water treatment agent increased to 400 mg/L, the discoloration time of the copper sulfate liquid drop on the surface of the carbon steel also exhibited a slight increase and the discoloration time increased to 36.51 s. Then, with crease in the concentration of polyamine water treatment agent, the discoloration time of the copper sulfate liquid drop on the surface of the carbon steel exhibited a slight decrease and decreased to 26.87 s. The results indicated that the corrosion resistance of carbon steel was improved significantly as the concentration of the polyamine water treatment agent increased to 100 mg/L, and then improved slightly as the concentration of the polyamine water treatment agent increased to 400 mg/L, and was finally unchanged with the further increase in the concentration of polyamine water treatment agent. Moreover, when the concentration of polyamine water treatment agent was 1000 mg/L, a thick granular substance was formed on the surface of the carbon steel; additionally, the discoloration time of the different copper sulfate liquid drops on the surface of the carbon steel exhibited great differences which indicated that the uniformity of the protective film on the surface of the carbon steel was poor.

Additionally, the elemental compositions of the carbon steel which was dipped in the solution with 10 mg/L of polyamine water treatment agent at 50 °C were measured by EDS and the experiment results are shown in Table 1. Compared to the dipped carbon steel with the pristine carbon steel, it was found that the N element was detected on the surface of the carbon steel after being dipped in the aqueous solution with the polyamine water treatment agent, which proved the presence of OLDA on the surface of the carbon steel.

### 3.2. The Electrochemical Properties of Carbon Steel

#### 3.2.1. The Analysis of the Polarization Curve

**Figure 3:**
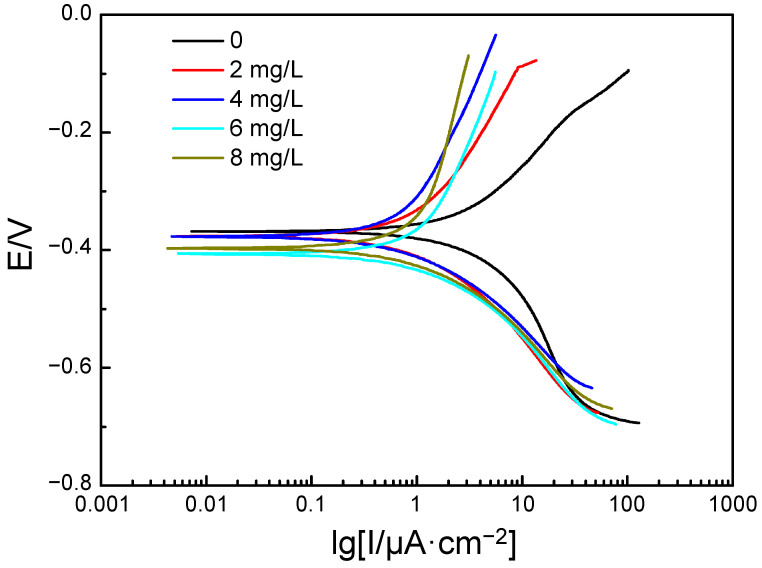
The polarization curves of carbon steel in 200 mg/L NaCl solution with different concentrations of the polyamine water treatment agent.

After soaking in aqueous solution with different concentrations of the polyamine water treatment agent for 24 h, the polarization curves of carbon steel were measured in the medium containing 200 mg/L of NaCl with the corresponding concentration of polyamine water treatment agent. The polarization curves are shown in Figure 3 and the fitting results of the polarization curves are shown in Table 2. As shown in Figure 3 and Table 2, the self-corrosion potential of the carbon steel exhibited no obvious difference but the self-corrosion current exhibited obvious changes under different concentrations of the polyamine water treatment agent. With the increase in concentration of polyamine water treatment agent, the self-corrosion current decreased at first, and then increased slightly. According to the changes in self-corrosion potential and self-corrosion current, it was seen that the polyamine water treatment agent exhibited a “geometric covering effect” on the carbon steel to reduce the corrosion; that is, the polyamine water treatment agent was adsorbed on the surface of the carbon steel to form a protective film, which could inhibit both the anode and cathode reaction, thus mitigating the corrosion degree of the carbon steel in NaCl solution. The polyamine water treatment agent exhibited a good corrosion inhibition effect on the carbon steel with a corrosion inhibition efficiency (η) of more than 50% calculated by self-corrosion current according to the fitting results of the polarization curves. With the increase in the concentration of the polyamine water treatment agent, the corrosion inhibition efficiency of the polyamine water treatment agent on the carbon steel increased at first, and then decreased slightly. When the concentration of polyamine water treatment agent was 4 mg/L, the corrosion inhibition effect of the polyamine water treatment agent on carbon steel was the best and the corrosion inhibition efficiency was 76.88%. In addition, the linear polarization resistances of the carbon steel under different conditions were obtained according to the polarization curves, which were 29.8, 89.6, 110.3, 79.2 and 91.2 kΩ·cm^2^ as the concentrations of polyamine water treatment agent were 0, 2, 4, 6 and 8 mg/L. Therefore, the corrosion inhibition efficiency could also be calculated from the linear polarization resistance [25]. The corrosion inhibition efficiency of the polyamine water treatment agent were 66.66%, 72.91%, 62.26% and 67.24%, which also indicated that the corrosion inhibition effect was the best as the concentration of polyamine water treatment agent was 4 mg/L. The corrosion inhibition efficiencies calculated by both self-corrosion current and linear polarization resistance all reflected 4 mg/L of polyamine water treatment agent could achieve the best corrosion inhibition effect. According to the two evaluated methods, it was observed that the corrosion inhibition effect of the polyamine water treatment agent on carbon steel exhibited extreme-concentration phenomenon, which might because that the film-forming amine contained both hydrophilic groups and hydrophobic groups. The micelles might be formed when the concentration of polyamine water treatment agent was high, resulting in a decrease in adsorption of the polyamine water treatment agent on the surface of the carbon steel and thus, a decline in the corrosion inhibition effect on carbon steel.

#### 3.2.2. The Analysis of Electrochemical Impedance Spectroscopy

**Figure 4:**
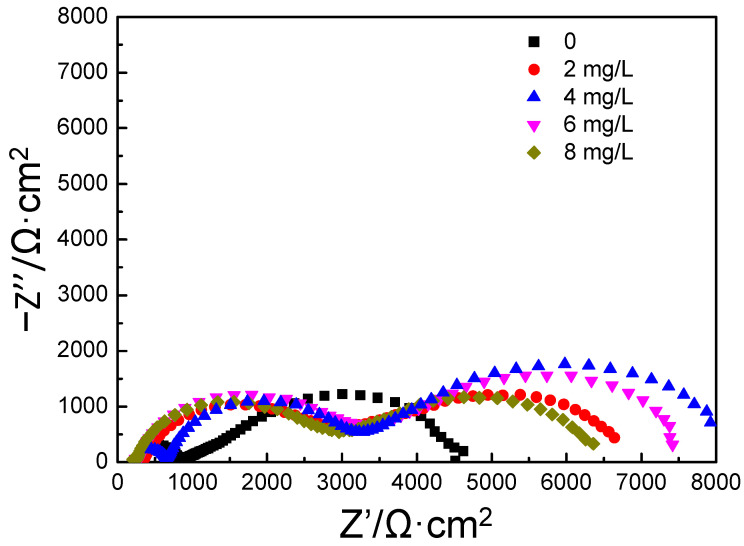
EIS spectra of carbon steel in 200 mg/L of NaCl solution with different concentrations of the polyamine water treatment agent.

After soaking in aqueous solution with different concentrations of the polyamine water treatment agent for 24 h, the EIS of carbon steel was measured in the medium containing 200 mg/L of NaCl with the corresponding concentration of polyamine water treatment agent. The Nyquist diagram is shown in Figure 4. When the concentration of the polyamine water treatment agent was 0, two arcs appeared in the Nyquist diagram of carbon steel, of which the left arc was a high frequency capacitive reactance arc and the right arc was a relatively large low frequency capacitive reactance arc. According to the characteristics of low frequency capacitive reactance arc, it was inferred that the electrode process of carbon steel was caused by the finite layer diffusion impedance. When the aqueous solution contained the polyamine water treatment agent, the Nyquist diagram of carbon steel exhibited obvious changes and three arcs appeared; that is, a new arc was formed in the high–middle frequency region in addition to the high frequency capacitive reactance arc and the low frequency finite layer diffusion impedance arc. The electrode process of carbon steel was not only affected by the electrode potential, but also affected by the film-forming coverage of the polyamine water treatment agent on its surface; moreover, the protective film formed by the adsorption of the polyamine water treatment agent on the surface of carbon steel exhibited capacitive impedance. Therefore, the additional new capacitive arc was formed in the Nyquist diagram of carbon steel. In addition, the radius of the original high frequency capacitive reactance arc decreased and the radius of the low frequency finite layer diffusion impedance arc increased at first, and then decreased with the increase in the concentration of polyamine water treatment agent.

**Figure 5:**
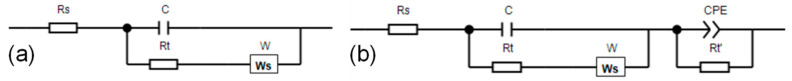
The equivalent circuit of EIS of the carbon steel in 200 mg/L of NaCl solution (**a**) without the polyamine water treatment agent and (**b**) with the different concentrations of the polyamine water treatment agent.

**Figure 6:**
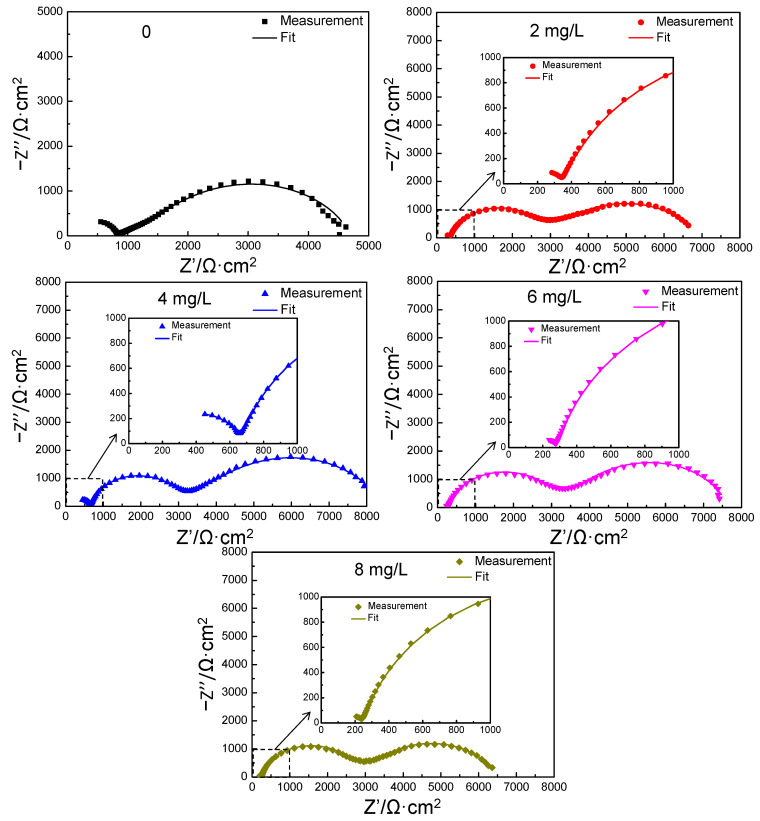
The fitting curve and EIS of the carbon steel in 200 mg/L of NaCl solution with different concentrations of the polyamine water treatment agent.

The equivalent circuits of the EIS of carbon steel in the medium containing 200 mg/L of NaCl without the polyamine water treatment agent and with the different concentrations of the polyamine water treatment agent are shown in Figure 5a,b. R_s_ was the solution resistance between the working electrode and the reference electrode, R_t_ was the charge transfer resistance (charged particles in solution passed through the electric double layer on the surface of the working electrode), C was the electric double layer capacitance on the surface of electrode, W was the finite layer diffusion impedance, R_t_’ was the charge transfer resistance of the working electrode with adsorbed polyamine water treatment agent on the surface, CPE was the constant phase angle element of the working electrode with adsorbed polyamine water treatment agent on the surface. The fitting curves of the electrochemical impedance according to the equivalent circuit are shown in Figure 6 and it was found that the fitting curves were consistent with the measurements of the electrochemical impedance of carbon steel under different conditions. The fitting values of different components of the equivalent circuit are shown in Table 3. When the aqueous solution contained the polyamine water treatment agent, the solution resistance R_s_ decreased slightly because the addition of the polyamine water treatment agent increased the conductivity of the aqueous solution. With the increase in the concentration of polyamine water treatment agent, the charge transfer resistance R_t_ increased at first, and then decreased, and the finite layer diffusion resistance R and capacitance Y_0_ increased at first, and then decreased. When the aqueous solution contained the polyamine water treatment agent, the new added constant phase angle element Y_0′_ decreased at first, and then increased, and n′ increased at first and then decreased. When the concentration of polyamine water treatment agent was 4 mg/L, the constant phase angle element Y_0′_ exhibited the smallest value and n′ exhibited the largest value, which suggested that the protective film formed by the polyamine water treatment agent on the surface of carbon steel was the thickest and showed the best uniformity. In addition, according to the fitting results of the equivalent circuits, the total resistance value R_total_ increased at first, and then decreased, suggesting that the corrosion inhibition effect of film-forming amine increased at first, and then declined. When the concentration of polyamine water treatment agent was 4 mg/L, the corrosion inhibition effect was the best and the corrosion inhibition rate was 82.30%. The EIS results indicated that the corrosion inhibition effect of the polyamine water treatment agent on carbon steel exhibited the extreme-concentration phenomenon, which was consistent with the results of the polarization curves.

### 3.3. The Corrosion Inhibition Effect of Polyamine Water Treatment Agent

#### 3.3.1. pH Regulation Performance of Polyamine Water Treatment Agent

**Figure 7:**
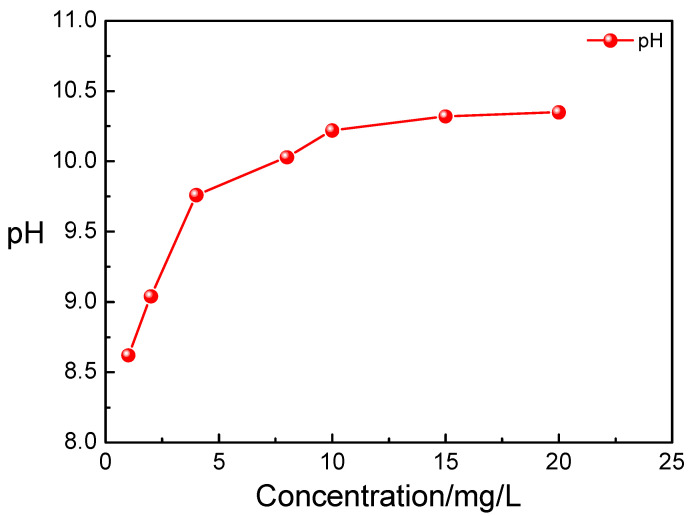
pH of the pure water with different concentrations of the polyamine water treatment agent.

pH regulation performance of a boiler water treatment agent is an important property. During the operation of a boiler water treatment system, a type of boiler water treatment agent was added to regulate the pH of boiler water to reduce the metal corrosion. The pH regulation performance of the polyamine water treatment agent was measured and is shown in Figure 7. When the polyamine water treatment agent was added into the pure water, the pH of pure water increased with the increase in the concentration of the polyamine water treatment agent. As the concentration of the polyamine water treatment agent was 1~4 mg/L, the pH of pure water increased significantly with the increase in the concentration of polyamine water treatment agent. As the concentration of polyamine water treatment agent was above 4 mg/L, the pH of pure water increased slightly with the increase in the concentration of polyamine water treatment agent. And as the concentration of polyamine water treatment agent was above 10 mg/L, the pH of pure water was not almost changed with the increase in the concentration of polyamine water treatment agent. During the actual operation of a boiler, the pH values of the boiler feed water and furnace water were generally maintained at 8.8~9.6 and 9.2~9.7, respectively, according to the standard. The prepared polyamine water treatment agent could be used as the boiler water treatment agent to adjust the pH of pure water according to the standard; additionally, the concentration of the needed polyamine water treatment agent was low.

#### 3.3.2. The Corrosion Inhibition Effect of Polyamine Water Treatment Agent on Carbon Steel

The corrosion rates of carbon steel in the liquid and gas phase environments of pure water without and with the polyamine water treatment agent at different temperatures were measured and are shown in Table 4. According to the electrochemical analyses results and the pH regulation performance of different concentrations of the polyamine water treatment agent, the concentration of polyamine water treatment agent in pure water was 4 mg/L. At 150 °C, the corrosion rates of the carbon steel in the liquid and gas phase environments of pure water were 0.06985 and 0.05456 mm/a, and at 350 °C, the corrosion rates of the carbon steel in liquid and gas phase environments of pure water increased to 0.1298 and 0.08679 mm/a, respectively. According to the results, the increase in temperature contributed more to the increase in the corrosion rate of carbon steel in the liquid phase environment than in the gas phase environment of pure water. 

**Figure 8:**
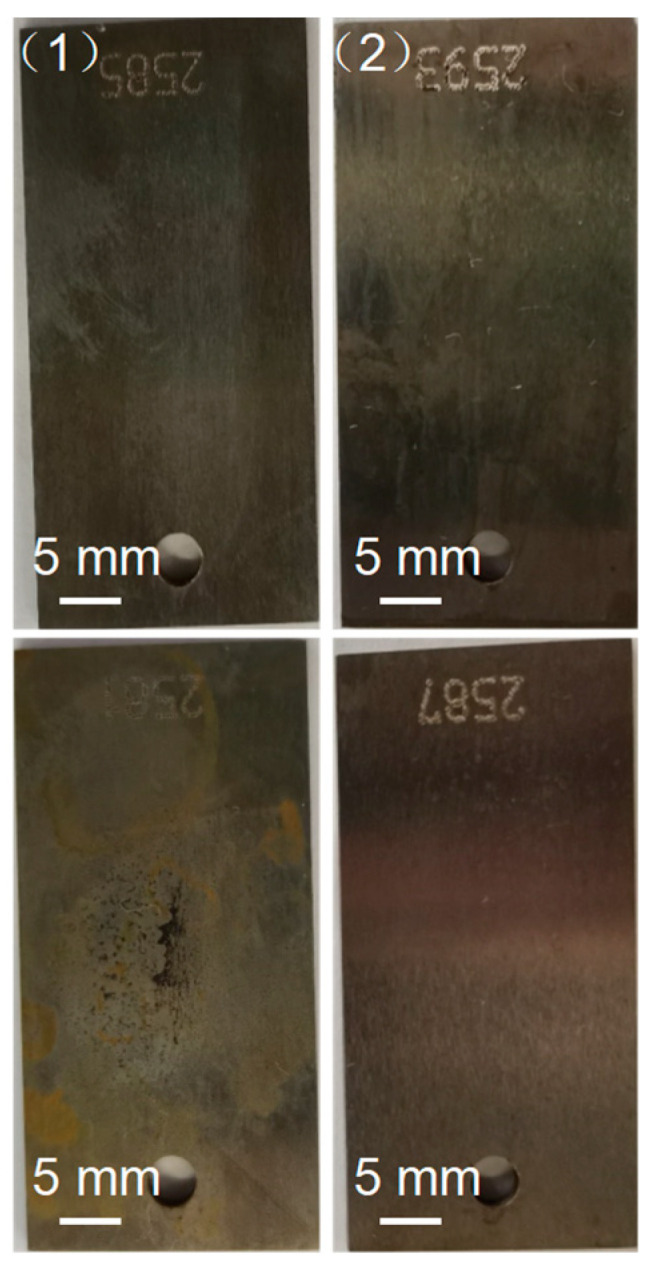
The pictures of the corroded carbon steel in (**up**) liquid and (**down**) gas phase environments of pure water (1) without and (2) with the polyamine water treatment agent at 150 °C after cleaning.

When the polyamine water treatment agent was added to the pure water, the corrosion rates of carbon steel in liquid and gas environments exhibited a varying rate of decrease. At 150 °C, the corrosion rates of carbon steel in the liquid and gas phase environments of pure water with the polyamine water treatment agent decreased to 0.03224 and 0.01777 mm/a. According to the corrosion rate, the corrosion inhibition rates of the polyamine water treatment agent on carbon steel in liquid and gas environments were 53.84% and 67.43%, respectively. The results indicated that the polyamine water treatment agent could not only form the protective film on the surface of carbon steel immersed in the liquid phase, but also formed the protective film on the surface of carbon steel in the gas phase, thereby reducing the corrosion of carbon steel in a steam–water system. The photos of the corroded carbon steel in liquid and gas phases of pure water without and with the polyamine water treatment agent after cleaning are shown in Figure 8. It was observed that the surface of the corroded carbon steel in liquid and gas phases of pure water without and with the polyamine water treatment agent exhibited no obvious difference. Since at low temperature the corrosion rate of carbon steel was small, the surface of carbon steel exhibited no obvious changes after corroded and it was difficult to observe the differences between different corroded carbon steels.

**Figure 9:**
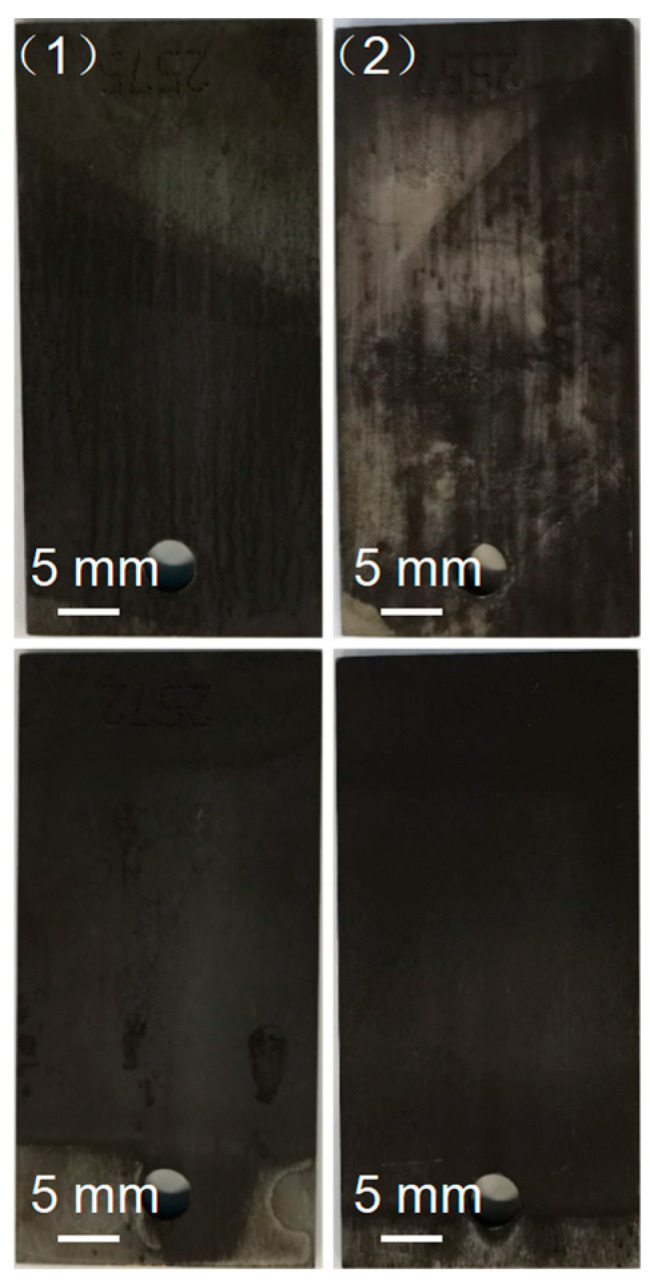
The pictures of the corroded carbon steel in (**up**) liquid and (**down**) gas phase environments of pure water (1) without and (2) with the polyamine water treatment agent at 350 °C after cleaning.

When the experiment temperature was 350 °C, the corrosion rates of carbon steel in liquid and gas phase environments of pure water with the polyamine water treatment agent were 0.09091 and 0.08639 mm/a, respectively. It was observed that when the polyamine water treatment agent was added into the pure water, the corrosion rates of carbon steel in liquid phase environment decreased significantly but the corrosion rate of carbon steel in gas phase environment was almost unchanged. According to the corrosion rate results, the corrosion inhibition rates of the polyamine water treatment agent on carbon steel in liquid and gas environments were 29.96% and 0.46%, respectively. The results indicated that the polyamine water treatment agent exhibited a good corrosion inhibition effect on the carbon steel in the liquid phase environment while it exhibited almost no corrosion inhibition effect on the carbon steel in the gas phase environment. The photos of the corroded carbon steel in liquid and gas phases of pure water without and with the polyamine water treatment agent after cleaning are shown in Figure 9. The surface of the corroded carbon steel in the liquid and gas environments of pure water exhibited no obvious differences and became black. When the polyamine water treatment agent was added into the pure water, the surface of the corroded carbon steel in the liquid environment was still black but the black materials could be partly removed by cleaning, and the surface of the corroded carbon steel in the gas environment was the same as that in the gas environment of pure water. According to the surface images of the corroded carbon steel, it was also found that the polyamine water treatment agent exhibited a good corrosion inhibition effect on the carbon steel in the liquid phase environment, while almost no corrosion inhibition effect was identified on the carbon steel in the gas phase environment.

According to the corrosion experiment of carbon steel in a steam–water system, it was seen that as the polyamine water treatment agent was added into the pure water, the corrosion of carbon steel in both the liquid and gas environments was mitigated differently. Combined with the film-forming and electrochemical properties, it was concluded that the polyamine water treatment agent could form a protective film on the surface of carbon steel, which hindered the diffusion and migration of corrosive media such as water molecules or oxygen to the surface of carbon steel, and also hindered the dissolution and diffusion of metal ions; thereby, the anode and cathode reactions required for carbon steel corrosion were inhibited and this reduced the corrosion of carbon steel in the liquid phase environment. For the carbon steel in the gas phase environment, the volatilized polyamine water treatment agent could also form a protective film on the surface of carbon steel due to the high temperature although the polyamine water treatment agent was added into the liquid phase of pure water, which reduced the chemical reaction rate between water vapor, oxygen, etc., and iron, thereby reducing the corrosion of carbon steel in gas phase environment.

#### 3.3.3. Surface Micromorphology of Carbon Steel after Corrosion Experiment

**Figure 10:**
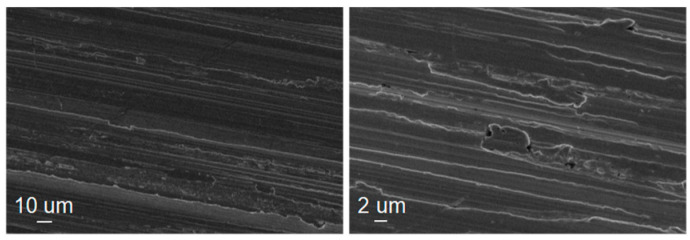
SEM of the pristine carbon steel.

**Figure 11:**
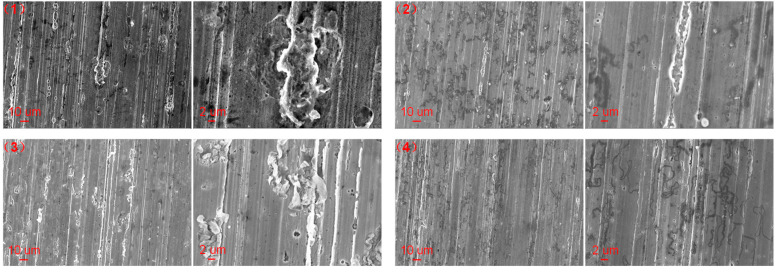
SEM of the corroded carbon steel in (**up**) liquid and (**down**) gas phase environments of pure water (1), (3) without and (2), (4) with the polyamine water treatment agent at 150 °C after cleaning.

**Figure 12:**
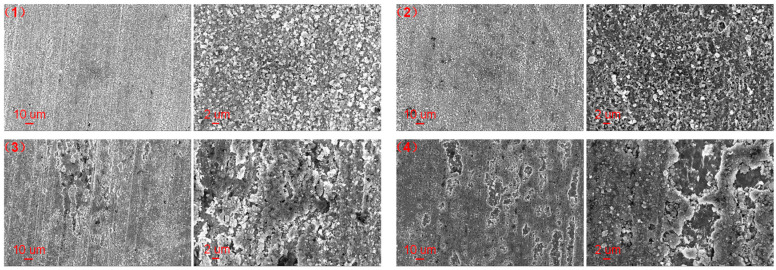
SEM of the corroded carbon steel in (**up**) liquid and (**down**) gas phase environments of pure water (1), (3) without and (2), (4) with the polyamine water treatment agent at 350 °C after cleaning.

To further study the corrosion of carbon steel under different conditions, the surface micromorphology of carbon steel was characterized and is shown in Figure 10, Figure 11 and Figure 12 and the elemental compositions on the surface of different carbon steels were measured and shown in Table 5. As shown in Figure 10, the surface of the pristine carbon steel was relatively flat and textured. The surface elements of the pristine carbon steel were mainly composed of Fe and C, and the mass contents of Fe and C were 95.9% and 4.1%, respectively. The surface micromorphology of the corroded carbon steel in the liquid and gas phase environments of pure water without and with the polyamine water treatment agent at 150 °C are shown in Figure 11. The surface of the corroded carbon steel in the liquid and gas phase environments of pure water was attached with the clump substance. As shown in Table 5, O element appeared on the surface of the corroded carbon steel and the mass contents of Fe, C and O of the corroded carbon steel in liquid and gas environments were 89.87%, 4.24% and 5.89% and 93.16%, 3.07% and 3.77%, respectively. When the polyamine water treatment agent was added into the pure water, the surface of the corroded carbon steel in the liquid and gas phase environments was similar to that of the pristine carbon steel and there were no clump substances. But the O element also appeared on the surface of the corroded carbon steel and the mass contents of Fe, C and O of the corroded carbon steel in the liquid and gas environment were 92.47%, 2.86% and 4.67% and 4.13%, 3.28% and 2.59%, respectively. According to the elemental compositions, it was found that the oxygen content of the corroded carbon steel in pure water with the polyamine water treatment agent was lower than that without the polyamine water treatment agent, which indicated that the addition of the polyamine water treatment agent in pure water reduced the formation of corrosion products on the carbon steel.

The surface micromorphology and elemental compositions of the corroded carbon steel in liquid and gas phase environments of pure water without and with the polyamine water treatment agent at 350 °C are shown in Figure 12 and Table 5. The surface of the corroded carbon steel in the liquid and gas phase environments of pure water exhibited obvious differences from that of the pristine carbon steel and was composed of particles with a regular shape. The mass contents of Fe, C and O of the corroded carbon steel in liquid and gas environments of pure water were 71.61%, 2.73% and 25.67% and 72.79%, 2.97% and 24.24%, respectively. It was seen that the oxygen content of the corroded carbon steel was high and it was inferred that the particles with a regular shape were iron oxide. When the polyamine water treatment agent was added into the pure water, the surface micromorphology of the corroded carbon steel was similar to that in pure water but the oxygen content of the corroded carbon steel declined significantly. The mass contents of Fe, C and O of the corroded carbon steel in the liquid and gas environments of pure water with the polyamine water treatment agent were 86.20%, 3.07% and 10.74% and 84.86%, 5.05% and 10.08%, respectively. It was found that the iron oxide on the corroded carbon steel was reduced significantly when the polyamine water treatment agent was added into the pure water, indicating that there was a good corrosion inhibition effect of the polyamine water treatment agent on the carbon steel.

## 4. Conclusions

A polyamine water treatment agent was prepared by dissolving OLDA into CHA which was regarded as a hydrotrope to solve the problem that OLDA could not be soluble in water. The film-forming properties of the polyamine water treatment agent on the carbon steel were characterized by the copper sulfate liquid drop experiment. The corrosion inhibition mechanism of the polyamine water treatment agent on the carbon steel was analyzed by the electrochemical characterization, including polarization curves and electrochemical impedance spectroscopy. The corrosion inhibition effect of the polyamine water treatment agent on the carbon steel in a boiler steam–water system was evaluated with a metal corrosion experiment in an autoclave at different temperatures and the surface micromorphology of the corroded carbon steel was further characterized.

The results indicated the polyamine water treatment agent could form a protective film on the carbon steel to improve the corrosion inhibition. The protective film on the carbon steel formed by the polyamine water treatment agent exhibited a geometric effect, which could inhibit both the anode and cathode reaction of carbon steel, so as to reduce the corrosion of carbon steel in the medium. According to the polarization curves and electrochemical impedance spectrum analyses, the corrosion inhibition effect of the polyamine water treatment agent on carbon steel exhibited the extreme-concentration phenomenon. The polyamine water treatment agent in pure water reduced the corrosion of carbon steel in a simulated boiler steam–water system. The corrosion inhibition effect of the polyamine water treatment agent on carbon steel at 150 °C was better than that at 350 °C, and the corrosion inhibition rates of the polyamine water treatment agent on carbon steel in liquid and gas environments at 150 °C were 53.84% and 67.43%, respectively. SEM-EDS characterization indicated that the polyamine water treatment agent reduced the formation of the corrosion product, iron oxide, on the carbon steel in the simulated boiler steam–water system. 

## Figures and Tables

**Table 1:** The element compositions on the surface of the different carbon steels according to EDS measurements.

Carbon Steel Sample	Element Mass Content		
	Fe/%	C/%	N/%
Pristine carbon steel	95.90	4.10	/
Dipped carbon steel	93.51	5.78	0.71

**Table 2:** The fitting results of the polarization curves of carbon steel in 200 mg/L of NaCl solution with different concentrations of the polyamine water treatment agent.

C/mg/L	I_corr_/μA/cm^2^	−E_corr_/V	B_a_/mV	B_c_/mV	η/%
0	4.4223	0.3682	232.43	306.43	
2	1.4717	0.3764	367.16	205.92	66.72
4	1.0222	0.3771	478.3	147.57	76.88
6	1.8282	0.4067	697.8	182.69	58.66
8	1.8864	0.3968	2160	186.98	57.34

**Table 3:** The fitting results of EIS of the carbon steel in 200 mg/L of NaCl solution with different concentrations of the polyamine water treatment agent.

C/mg/L	R_s_/Ω·cm^2^	C/F·cm^−2^	R_t_/Ω·cm^2^	W			CPE		R_t_′/Ω·cm^2^	R_total_/Ω·cm^2^	η/%
				R/Ω·cm^2^	Y_0_/F·cm^−2^	n	Y_0′_/F·cm^−2^	n′			
0	200.17	2.34 × 10^−9^	608.97	3898.6	3.3406	0.39138				4507.57	
2	49.182	1.94 × 10^−9^	255.42	4492.8	4.409	0.36323	1.62 × 10^−6^	0.90959	2113.1	6861.32	52.22
4	167.77	2.80 × 10^−9^	465.09	5649.3	6.4494	0.40182	1.36 × 10^−6^	0.9429	2102.7	8217.09	82.30
6	99.358	3.86 × 10^−9^	159.01	4886.2	3.9901	0.41901	1.54 × 10^−6^	0.93016	2502.6	7547.81	67.45
8	93.242	4.32 × 10^−9^	127.4	4000.3	4.4644	0.39224	1.47 × 10^−6^	0.92947	2240.2	6367.9	41.27

**Table 4:** The corrosion rates of carbon steel in liquid and gas phase environments of pure water with different concentrations of the polyamine water treatment agent and the corrosion inhibition rate of polyamine water treatment agent.

Concentration/ mg/L	Temperature/ °C	Pressure/ MPa	Time/h	Medium	Corrosion Rate/mm/a			Average Corrosion Rate/mm/a	Corrosion Inhibition Rate/%
					1	2	3		
0	150	0	24	Liquid	0.06448	0.06696	0.07812	0.06985	/
				Gas	0.07068	0.05208	0.04092	0.05456	/
4	150	0	24	Liquid	0.03224	0.03472	0.02976	0.03224	53.84
				Gas	0.02232	0.01736	0.01364	0.01777	67.43
0	350	16.5	24	Liquid	0.1364	0.1240	0.1290	0.1298	/
				Gas	0.09547	0.08803	0.07688	0.08679	/
4	350	16.5	24	Liquid	0.07563	0.1066	0.09051	0.09091	29.96
				Gas	0.07315	0.08431	0.1017	0.08639	0.46

**Table 5:** The elemental compositions on the surface of different carbon steels according to EDS measurements (1 was the pristine carbon steel, 2 and 3 were the corroded carbon steels in liquid and gas phase environments of pure water at 150 °C, 4 and 5 were the corroded carbon steels in liquid and gas phase environments of pure water with the polyamine water treatment agent at 150 °C, 6 and 7 were the corroded carbon steels in liquid and gas phase environments of pure water at 350 °C, 8 and 9 were the corroded carbon steels in liquid and gas phase environments of pure water with the polyamine water treatment agent at 350 °C).

Carbon Steel Sample	Element Mass Content		
	Fe/%	C/%	O/%
1	95.90	4.10	/
2	89.87	4.24	5.89
3	93.16	3.07	3.77
4	92.47	2.86	4.67
5	94.13	3.28	2.59
6	71.61	2.73	25.67
7	72.79	2.97	24.24
8	86.20	3.07	10.74
9	84.86	5.05	10.08

## Data Availability

Data are contained within the article.
